# Optimizing subgroup selection in two‐stage adaptive enrichment and umbrella designs

**DOI:** 10.1002/sim.8949

**Published:** 2021-03-29

**Authors:** Nicolás M. Ballarini, Thomas Burnett, Thomas Jaki, Christoper Jennison, Franz König, Martin Posch

**Affiliations:** ^1^ Section for Medical Statistics Medical University of Vienna Vienna Austria; ^2^ Department of Mathematics and Statistics Lancaster University Lancaster UK; ^3^ MRC Biostatistics Unit University of Cambridge Cambridge UK; ^4^ Department of Mathematical Sciences University of Bath Bath UK

**Keywords:** Bayesian optimization, conditional error function, subgroup analysis, utility function

## Abstract

We design two‐stage confirmatory clinical trials that use adaptation to find the subgroup of patients who will benefit from a new treatment, testing for a treatment effect in each of two disjoint subgroups. Our proposal allows aspects of the trial, such as recruitment probabilities of each group, to be altered at an interim analysis. We use the conditional error rate approach to implement these adaptations with protection of overall error rates. Applying a Bayesian decision‐theoretic framework, we optimize design parameters by maximizing a utility function that takes the population prevalence of the subgroups into account. We show results for traditional trials with familywise error rate control (using a closed testing procedure) as well as for umbrella trials in which only the per‐comparison type 1 error rate is controlled. We present numerical examples to illustrate the optimization process and the effectiveness of the proposed designs.

## 1 INTRODUCTION

It is increasingly common to integrate subgroup identification and confirmation into a clinical development program. Biomarker‐guided clinical trial designs have been proposed to close the gap between the exploration and confirmation of subgroup treatment effects. Numerous statistical considerations (eg, multiplicity issues, consistency of treatment effects, trial design) need to be taken into account to ensure a proper interpretation of study findings, as outlined in recent reviews.^1^, ^2^, ^3^, ^4^

Several study designs are available for the investigation of subgroups in clinical trials. These include all‐comers designs where biomarker status or subgroup are not considered for enrolment but only in the trial analysis, and stratified designs where the trial prevalences for each subgroup, that is the proportion of patients recruited from each subgroup, are chosen initially and maintained throughout the trial.^5^, ^6^ Adaptive enrichment designs have been proposed to increase the efficiency of these trials.^7^, ^8^, ^9^, ^10^, ^11^ These designs allow subgroups to be dropped for futility at interim analyses with the rest of the trial being conducted with subjects from the remaining groups only. The U.S. Food and Drug Administration guidance on adaptive designs highlights the use of adaptive enrichment designs as a means to increase the chance to detect a true drug effect over that of a fixed sample design.^12^

Master protocols provide an infrastructure for efficient study of newly developed compounds or biomarker‐defined subgroups.^13^, ^14^ Such studies simultaneously evaluate more than one investigational drug or more than one disease type within the same overall trial structure.^15^, ^16^, ^17^ An umbrella trial is a particular type of master protocol in which enrolment is restricted to a single disease but the patients are screened and assigned to molecularly defined subtrials. Each subtrial may have different objectives, endpoints or design characteristics. An example of an umbrella trial is the ALCHEMIST trial, in which patients with nonsmall cell lung cancer are screened for EGFR mutation or ALK rearrangement and assigned accordingly to subtrials with different treatments.^18^

In this paper, we study confirmatory trials that allow the investigation of the treatment effect in prespecified nonoverlapping subgroups. In particular, we focus on adaptive clinical trials that allow the modification of design elements without compromising the integrity of the trial.^19^ We propose a class of adaptive enrichment designs that use a Bayesian decision framework to optimize the design parameters, such as the trial prevalences of the subgroups, the weights for multiple hypotheses testing, and adaptation rules. A similar framework has been used in References 20, 21, 22, 23, 24, 25, 26, 27 for adaptive enrichment trials.

We consider two types of problem. In the first case, we study designs that preserve the familywise error rate (FWER) of the trial using a closed testing procedure to test the null hypotheses of no treatment effect in the two subgroups. This is what is typically required in adaptive enrichment trials where a single treatment is evaluated against a control. In the second case, we show results for umbrella trial designs without multiplicity adjustment. Here, we consider studies made up of separate simultaneous trials, for which it has been argued that no control of multiplicity is needed.^28^ Our work, therefore, provides an overarching framework for both adaptive enrichment designs and umbrella trials.

The manuscript is organized as follows: In Section 2, we introduce the designs and distinguish between single‐stage designs (Section 2.2) and two‐stage designs (Section 2.3), and in Section 2.4 we discuss how to adapt our proposed designs to umbrella trials. In Sections 3 and 4 we present numerical examples. We describe how our methods may be extended to designs with more than two stages in Section 5 and we end with conclusions and a discussion in Section 6.

## 2 BAYES OPTIMAL DESIGNS

### 2.1 The class of trial designs

Consider a confirmatory parallel‐group clinical trial comparing a new treatment and a control with respect to a pre‐defined primary endpoint. We assume the patient population may be divided into disjoint, biomarker‐defined subgroups. Given a maximum achievable sample size, *n*, we aim to optimize the trial design by maximising a specific utility function.

Suppose two biomarker‐defined subgroups have been identified before commencing the trial. Let 0<λ<1 be the prevalence of the first subgroup in the underlying patient population and 1−λ the prevalence of the second subgroup. Let $\theta_{1}$ and $\theta_{2}$ be the treatment effects, denoting the difference in the mean outcome between treatment and control, in the first and second subgroups, respectively. We consider trials to investigate the null hypotheses *H*_01_: $\theta_{1}\leq 0$ and *H*_02_: $\theta_{2}\leq 0$ with corresponding alternative hypotheses *H*_11_: $\theta_{1}>0$ and *H*_12_: $\theta_{2}>0$. In Sections 2.2 and 2.3 we consider confirmatory trials in which strong control of the FWER is imposed.^29^ In our discussion of umbrella trials in Section 2.4, we assume multiplicity control is not required.

**FIGURE 1:**
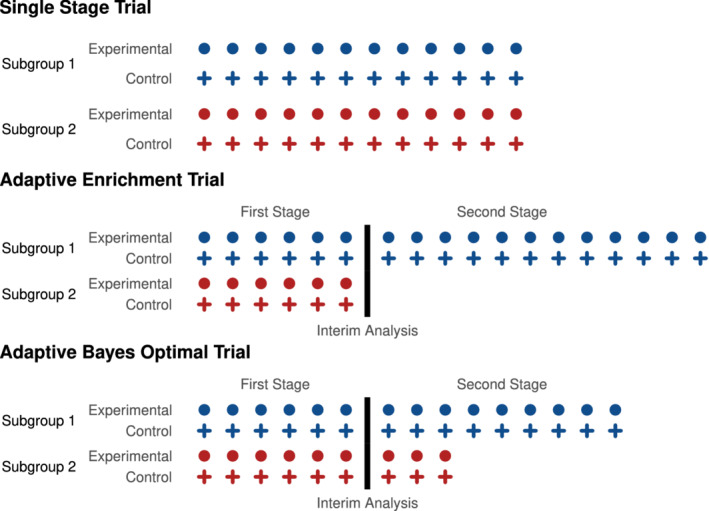
Schematic representation of the three types of trial design. In the single‐stage trial, the sampling prevalences of the subgroups are fixed throughout the trial. In standard adaptive enrichment trials, patients are recruited with predefined subgroup prevalences until the interim analysis, at which point a decision is taken to continue with the same prevalences or to sample from a single subgroup. In the Bayes optimal adaptive trial designs that we consider, the sampling prevalences may be changed at the interim analysis [Colour figure can be viewed at wileyonlinelibrary.com]

We consider optimization within a class of designs 𝒜 that have a single interim analysis at which adaptation can take place. The total sample size is fixed at *n* with *s*^(1)^*n* patients in the first stage and *s*^(2)^*n* patients in the second stage, where *s*^(1)^ > 0, *s*^(2)^ ≥ 0 and *s*^(1)^ + *s*^(2)^ = 1. In the first stage, $r_{1}^{\left(1\right)}s^{\left(1\right)}n$ patients are recruited from subgroup 1 and $r_{2}^{\left(1\right)}s^{\left(1\right)}n$ from subgroup 2, where $r_{1}^{\left(1\right)}\geq 0$, $r_{2}^{\left(1\right)}\geq 0$ and $r_{1}^{\left(1\right)}+r_{2}^{\left(1\right)}=1$. In the second stage, $r_{1}^{\left(2\right)}s^{\left(2\right)}n$ patients are recruited from subgroup 1 and $r_{2}^{\left(2\right)}s^{\left(2\right)}n$ from subgroup 2, where $r_{1}^{\left(2\right)}\geq 0$, $r_{2}^{\left(2\right)}\geq 0$ and $r_{1}^{\left(2\right)}+r_{2}^{\left(2\right)}=1$, and the values of $r_{1}^{\left(2\right)}$ and $r_{2}^{\left(2\right)}$ may depend on the first stage data. Within each stage and subgroup, we assume equal allocation to the two treatment arms (this assumption is not strictly necessary and could be relaxed). Figure 1 gives a schematic representation of the trial design.

The definition of a particular design in 𝒜 is completed by specifying the multiple testing procedure to be used and the method for combining data across stages when adaptation occurs. We use a closed testing procedure to control FWER, applying a weighted Bonferroni procedure to test the intersection hypothesis. In this procedure, weights are initially set as $\omega_{1}^{\left(1\right)}$ and $\omega_{2}^{\left(1\right)}$ but these may be modified in the second stage if adaptation occurs. The error rate for each hypothesis test is controlled by preserving the conditional type I error rate when an adaptation is made. Thus, while we use a Bayesian approach to optimize the design, the trial is analyzed using frequentist procedures that control error rates at the desired level, adhering to conventional regulatory standards.

We follow a Bayesian decision theoretic approach to optimize over trial designs in the class 𝒜. In assessing each design, we assume a prior distribution for the treatment effects in each subgroup and a utility function^30^ that quantifies the value of the trial's outcome. We shall optimize designs with respect to the timing of the interim analysis, the proportion of patients recruited from the two subgroups at each stage of the trial, the weights in the weighted Bonferroni test, and the rule for updating these weights given the interim data.

We summarize the data observed during the trial by the symbol $\hat{\theta }$, noting that this summary should contain information about the numbers of observations from each subgroup and weights to be used in the weighted Bonferroni test at each stage, as well as estimates of $\theta_{1}$ and $\theta_{2}$ obtained from observations before and after the interim analysis. We define our utility function to be

(1) $$𝒰(\hat{\theta })=\lambda \;1(\text{Reject}\;H_{01})+(1-\lambda )\;1(\text{Reject}\;H_{02}),$$

where 1(.) is the indicator function. By definition, the data summary $\hat{\theta }$ contains the information needed to determine if each of the hypotheses *H*_01_ and *H*_02_ is rejected.

The utility (1) involves the size of the underlying subgroups as well as the rejection of the corresponding hypotheses. Thus, rejection of the null hypothesis for a larger subgroup is given greater weight. If the population prevalence of the two subgroups is not known, a prior on λ may be added. We note that terms in the function (1) are positive when a null hypothesis is rejected but the associated treatment effect is very small or even negative: this issue could be addressed by multiplying each term by an indicator variable which takes the value 1 if the relevant parameter, $\theta_{1}$ or $\theta_{2}$, is larger than zero or above a clinically relevant threshold (eg, Stallard et al^31^ where a similar approach is used for treatment selection).

Since the trial design is optimized with respect to the stated utility, it is important to choose a utility function that reflects accurately the relative importance of possible trial outcomes. Furthermore, the definition of utility can be adapted to reflect the interest of different stakeholders, for example, Ondra et al^21^ and Graf, Posch and König^24^ propose utility functions that represent the view of a sponsor or take a public health perspective.

Let π(θ) denote the prior distribution for $\theta =(\theta_{1},\theta_{2})$. Then, the Bayes expected utility for a trial design a∈𝒜 is

$$W_{\pi (\theta )}(a)={𝔼}_{\pi (\theta )}\left[{𝔼}_{\theta }\left\{𝒰(\hat{\theta })\right\}\right],$$

where we have taken the expectation over the sampling distribution of the trial data given the true treatment effects θ, with an outer integral over the prior distribution π(θ).

When choosing the prior π(θ), it is important to remember that $W_{\pi (\theta )}(a)$ represents the expected utility, averaged over θ∼π(θ). If an “uninformative” prior is chosen, this will place weight on extreme scenarios, such as large negative treatment effects, which have little credibility. Thus, when considering the Bayes optimal design, it is important to use subjective, informative priors. In some cases, pilot studies or historic observational data may be available to construct the prior distribution.

In this paper, we assume the prior distribution π(θ) to be bivariate normal,

(2) $$\left(\begin{array}{c} \theta_{1}\\ \theta_{2} \end{array}\right)∼N\left(\left(\begin{array}{c} \mu_{1}\\ \mu_{2} \end{array}\right),\left(\begin{array}{cc} \psi_{1}^{2} & \rho \psi_{1}\psi_{2}\\ \rho \psi_{1}\psi_{2} & \psi_{2}^{2} \end{array}\right)\right).$$

Here, the correlation coefficient ρ reflects the belief about the existence of common factors that contribute to the treatment effects in the two subgroups.

### 2.2 Bayes optimal single‐stage design

#### 2.2.1 Patient recruitment and estimation

Suppose we wish to conduct a single‐stage trial, which is the special case where *s*^(2)^ = 0, usually referred to as a stratified design. For simplicity of notation in this section, we write *r*_*j*_and $\omega_{j}$ rather than $r_{j}^{\left(1\right)}$ and $\omega_{j}^{\left(1\right)}$ for *j* = 1 and 2. We assume patients can be recruited at these rates regardless of the true proportions λ and 1−λ in the underlying patient population. In addition, we assume that patients are randomised between the new treatment and the control with a 1 : 1 allocation ratio in each subgroup.

During the trial we observe a normally distributed endpoint for each patient and we assume a constant variance for all observations. For patient *i* from subgroup *j* on the new treatment we have $X_{ji}∼N(\mu_{Tj},\sigma^{2})$, *i* = 1, … , *r*_*j*_*n*/2, and for patient *i* from subgroup *j* on the control treatment we have $Y_{ji}∼N(\mu_{Cj},\sigma^{2})$, *i* = 1, … , *r*_*j*_*n*/2. The estimate of the treatment effect $\theta_{j}=\mu_{Tj}-\mu_{Cj}$ in subgroup *j*, is

(3) $${\hat{\theta }}_{j}={\bar{X}}_{j}-{\bar{Y}}_{j}=\frac{1}{r_{j}n/2}\overset{r_{j}n/2}{\underset{i=1}{\sum }}X_{ji}-\frac{1}{r_{j}n/2}\overset{r_{j}n/2}{\underset{i=1}{\sum }}Y_{ji},\;j=1,2.$$

#### 2.2.2 Hypothesis testing in the single‐stage design

Consider the case *s*^(2)^ = 0 and 0 < *r*_1_ < 1. Then

$${\hat{\theta }}_{j}|\theta_{j}∼N\left(\theta_{j},\frac{4\sigma^{2}}{r_{j}n}\right),\;j=1,2,$$

and the corresponding *Z*‐values

$$Z_{j}=\frac{{\hat{\theta }}_{j}\sqrt{r_{j}n}}{2\sigma },\;j=1,2,$$

follow standard normal distributions under the null hypotheses *H*_01_ and *H*_02_.

We use a closed testing procedure to ensure strong control of the FWER at α level.^32^ To construct this, we require level α tests of *H*_01_: $\theta_{1}\leq 0$, *H*_02_: $\theta_{2}\leq 0$ and *H*_01_ ∩ *H*_02_: $\left\{\theta_{1}\leq 0\right\}\cap \left\{\theta_{2}\leq 0\right\}$. We reject *H*_01_ globally if the level α tests reject *H*_01_ and *H*_01_ ∩ *H*_02_. Similarly, we reject *H*_02_ globally if the level α tests reject *H*_02_ and *H*_01_ ∩ *H*_02_.

For the individual tests we reject *H*_01_ if $Z_{1}\geq \Phi^{-1}(1-\alpha )$ and *H*_02_ if $Z_{2}\geq \Phi^{-1}(1-\alpha )$. To test the intersection hypothesis, we use a weighted Bonferroni test: given predefined weights $\omega_{1}$ and $\omega_{2}$, where $\omega_{1}+\omega_{2}=1$, we reject *H*_01_ ∩ *H*_02_ if $Z_{1}\geq \Phi^{-1}(1-\omega_{1}\alpha )$ or $Z_{2}\geq \Phi^{-1}(1-\omega_{2}\alpha )$. The resulting closed testing procedure is equivalent to the weighted Bonferroni‐Holm test and will be generalised to adaptive tests in Section 2.3.

We note that the choice of a closed testing procedure is not restrictive in this setting since any procedure that gives strong control of the FWER may be written as a closed testing procedure.^22^, ^23^ Furthermore in the special cases *r*_1_ = 1 and *r*_2_ = 1, where the trial recruits from only one of the subgroups, just one subgroup is tested and only the test of the individual hypothesis is required. These cases are accommodated in our general class of designs by setting $\omega_{1}=1$ when *r*_1_ = 1 and $\omega_{2}=1$ when *r*_2_ = 1.

#### 2.2.3 Bayesian optimization

In the single‐stage trial we wish to optimize the trial prevalences of each subgroup, *r*_1_ and *r*_2_, and the weights in the Bonferroni‐Holm procedure, $\omega_{1}$ and $\omega_{2}$. Given the constraints *r*_1_ + *r*_2_ = 1 and $\omega_{1}+\omega_{2}=1$, we denote the set of parameters to optimize by $a=(r_{1},\omega_{1})$.

Let $f(\hat{\theta }|\theta ,a)$ denote the conditional distribution of $\left({\hat{\theta }}_{1},{\hat{\theta }}_{2}\right)$ given θ for design parameters *a*. The Bayes expected utility is given by

$${𝔼}_{\pi (\theta )}\left[{𝔼}_{\theta }\left[𝒰(\hat{\theta })\right]\right]=\int_{\theta }\int_{\hat{\theta }}𝒰(\hat{\theta })f(\hat{\theta }|\theta ,a)\pi (\theta )\;d\hat{\theta }\;d\theta .$$

The Bayes optimal design is given by the pair $a=(r_{1},\omega_{1})$ that maximises the Bayes expected utility of the trial, that is

$$\underset{a}{\text{argmax}}\int_{\theta }\int_{\hat{\theta }}𝒰(\hat{\theta })f(\hat{\theta }|\theta ,a)\pi (\theta )\;d\hat{\theta }\;d\theta .$$

Given our simple choices for the prior distribution and the utility function this integral may be computed directly (see Section S1.2 of Appendix S1). We find the Bayes optimal single‐stage trial by a numerical search over possible values of *a*.

### 2.3 Bayes optimal two‐stage adaptive design

#### 2.3.1 Adding a second stage

Consider now a two‐stage design in which data from the first stage inform adaptations in the second stage. The estimate of $\theta_{j}$ for subgroup *j* based on data collected in stage *k* is

(4) $${\hat{\theta }}_{j}^{\left(k\right)}={\bar{X}}_{j}^{\left(k\right)}-{\bar{Y}}_{j}^{\left(k\right)},\;j=1,2,\;k=1,2,$$

where ${\bar{X}}_{j}^{\left(k\right)}$ and ${\bar{Y}}_{j}^{\left(k\right)}$ are the mean responses in subgroup *j* in stage *k* for the treatment arm and control arm, respectively. Given the value of $\theta =(\theta_{1},\theta_{2})$, the first stage estimates are independent with distributions

$${\hat{\theta }}_{j}^{\left(1\right)}|\theta_{j}∼N\left(\theta_{j},\frac{4\sigma^{2}}{r_{j}^{\left(1\right)}s^{\left(1\right)}n}\right),\;j=1,2.$$

The trial prevalences, $r_{1}^{\left(2\right)}$ and $r_{2}^{\left(2\right)}$, of the two subgroups in the second stage are dependent on ${\hat{\theta }}_{1}^{\left(1\right)}$ and ${\hat{\theta }}_{2}^{\left(1\right)}$ but, conditional on $r_{1}^{\left(2\right)}$ and $r_{2}^{\left(2\right)}$, the second‐stage estimates are independent and conditionally independent of ${\hat{\theta }}_{1}^{\left(1\right)}$ and ${\hat{\theta }}_{2}^{\left(1\right)}$ with

$${\hat{\theta }}_{j}^{\left(2\right)}|r_{j}^{\left(2\right)},\theta_{j}∼N\left(\theta_{j},\frac{4\sigma^{2}}{r_{j}^{\left(2\right)}s^{\left(2\right)}n}\right),\;j=1,2.$$

#### 2.3.2 Hypothesis testing in the two‐stage adaptive design

There is a variety of approaches to test multiple hypotheses in a two‐stage adaptive design.^33^, ^34^, ^35^, ^36^ We shall use a closed testing procedure to ensure strong control of the FWER at level α, as we did for the single‐stage design in Section 2.2.2. In constructing level α tests of the null hypotheses *H*_01_, *H*_02_ and *H*_01_ ∩ *H*_02_ we employ the conditional error rate approach.^37^, ^38^ Based on a reference design and its predefined tests, we calculate the conditional error rate for each hypothesis and define adaptive tests which preserve this conditional error rate, thereby controlling the overall type I error rate.

Consider a reference design in which the trial prevalences of subgroups 1 and 2 and the weights in the weighted Bonferroni test of *H*_01_ ∩ *H*_02_ remain the same across stages, so $r_{j}^{\left(2\right)}=r_{j}^{\left(1\right)}$ and $\omega_{j}^{\left(2\right)}=\omega_{j}^{\left(1\right)}$ for *j* = 1 and 2. In the reference design, tests are performed by pooling the stage‐wise data within each subgroup and treatment arm, and using the conventional test statistics, as for the single‐stage test. For *j* = 1 and 2, the pooled estimate of $\theta_{j}$ across the two stages of the trial is

$${\hat{\theta }}_{j}^{\left(p\right)}=s^{\left(1\right)}\;{\hat{\theta }}_{j}^{\left(1\right)}+s^{\left(2\right)}\;{\hat{\theta }}_{j}^{\left(2\right)},$$

with corresponding *Z*‐value

$$Z_{j}^{\left(p\right)}=\frac{{\hat{\theta }}_{j}^{\left(p\right)}}{\sqrt{4\sigma^{2}/(r_{j}^{\left(1\right)}n)}},$$

and the null hypothesis *H*_0*j*_ is rejected at level α if $Z_{j}^{\left(p\right)}>\Phi^{-1}(1-\alpha )$. Let

$$Z_{j}^{\left(1\right)}=\frac{{\hat{\theta }}_{j}^{\left(1\right)}}{\sqrt{4\sigma^{2}/(r_{j}^{\left(1\right)}s^{\left(1\right)}n)}},\;j=1,2,$$

then the conditional distribution of $Z_{j}^{\left(p\right)}$ given the interim data is

$$Z_{j}^{\left(p\right)}|Z_{j}^{\left(1\right)},\theta_{j}∼N\left(\sqrt{s^{\left(1\right)}}Z_{j}^{\left(1\right)}+s^{\left(2\right)}\theta_{j}\frac{\sqrt{r_{j}^{\left(1\right)}n}}{2\sigma },\;s^{\left(2\right)}\right),$$

and the conditional error rates for the tests of *H*_0*j*_ are

(5) $$A_{j}=\mathbb{P}\left(Z_{j}^{\left(p\right)}>\Phi^{-1}(1-\alpha )\;|\;Z_{j}^{\left(1\right)},\theta_{j}=0\right),\;j=1,2.$$

Similarly, the conditional error rate for the test of *H*_01_ ∩ *H*_02_ is

(6) $$A_{12}=\mathbb{P}\left\{Z_{1}^{\left(p\right)}>\Phi^{-1}(1-\omega_{1}^{\left(1\right)}\alpha )\;\text{or}\;Z_{2}^{\left(p\right)}>\Phi^{-1}(1-\omega_{2}^{\left(1\right)}\alpha )\;|\;Z_{1}^{\left(1\right)},Z_{2}^{\left(1\right)},\theta_{1}=\theta_{2}=0\right\}.$$

See Section S1.1 of Appendix S1 for further details on the derivations of the conditional distributions.

In the adaptive design, if no adaptations are made at the interim analysis we apply the tests as defined for the reference design. Suppose now that adaptations are made and the trial prevalences in stage 2 are set to be $r_{1}^{\left(2\right)}$ and $r_{2}^{\left(2\right)}$ with weights $\omega_{1}^{\left(2\right)}$ and $\omega_{2}^{\left(2\right)}$ for the weighted Bonferroni test. In this case, we calculate the conditional error rates *A*_1_, *A*_2_ and *A*_12_ prior to adaptation from Equations (5) and (6). We then define tests of *H*_01_, *H*_02_ and *H*_01_ ∩ *H*_02_ based on stage 2 data alone that have these conditional error rates as their type 1 error probabilities. Given the updated $r_{1}^{\left(2\right)}$ and $r_{2}^{\left(2\right)}$,

$$Z_{j}^{\left(2\right)}\;|\;r_{j}^{\left(2\right)},\theta_{j}∼N\left(\theta_{j}\frac{\sqrt{r_{j}^{\left(2\right)}s^{\left(2\right)}n}}{2\sigma },\;1\right),\;j=1,2.$$

Thus, in our level α tests, we reject *H*_01_ if $Z_{1}^{\left(2\right)}>\Phi^{-1}(1-A_{1})$, we reject *H*_02_ if $Z_{2}^{\left(2\right)}>\Phi^{-1}(1-A_{2})$ and, applying a weighted Bonferroni test with weights $\omega_{1}^{\left(2\right)}$ and $\omega_{2}^{\left(2\right)}$, we reject *H*_01_ ∩ *H*_02_ if $Z_{1}^{\left(2\right)}>\Phi^{-1}(1-\omega_{1}^{\left(2\right)}A_{12})\;\text{or}\;Z_{2}^{\left(2\right)}>\Phi^{-1}(1-\omega_{2}^{\left(2\right)}A_{12})$. Finally, following the closed testing procedure, we reject *H*_01_ globally if the level α tests reject *H*_01_ and *H*_01_ ∩ *H*_02_ and we reject *H*_02_ globally if the level α tests reject *H*_02_ and *H*_01_ ∩ *H*_02_.

#### 2.3.3 Two‐stage optimization

We denote the set of initial design parameters by $a_{1}=(s^{\left(1\right)},r_{1}^{\left(1\right)},\omega_{1}^{\left(1\right)})$ and the second‐stage parameters by $a_{2}=(r_{1}^{\left(2\right)},\omega_{1}^{\left(2\right)})$. Let ${\hat{\theta }}^{\left(1\right)}=({\hat{\theta }}_{1}^{\left(1\right)},{\hat{\theta }}_{2}^{\left(1\right)})$ and ${\hat{\theta }}^{\left(2\right)}=({\hat{\theta }}_{1}^{\left(2\right)},{\hat{\theta }}_{2}^{\left(2\right)})$ be the vectors of estimated treatment effects in each subgroup, based on the first and second‐stage data, respectively, as defined in Equation (4). Denote the conditional distributions of the estimated effects in each stage of the trial by $f_{1}({\hat{\theta }}^{\left(1\right)}|\theta ,a_{1})$ and $f_{2}({\hat{\theta }}^{\left(2\right)}|\theta ,a_{2})$ and the posterior distribution of θ given the stage 1 observations by $\pi (\theta |{\hat{\theta }}^{\left(1\right)},a_{1})$. Then, the Bayes expected utility can be written as

(7) $${𝔼}_{\pi (\theta )}\left[{𝔼}_{\theta }\left[𝒰(\hat{\theta })\right]\right]=\int_{\theta }\int_{{\hat{\theta }}^{\left(1\right)}}\int_{{\hat{\theta }}^{\left(2\right)}}𝒰(\hat{\theta })f_{2}({\hat{\theta }}^{\left(2\right)}|\theta ,a_{2})f_{1}({\hat{\theta }}^{\left(1\right)}|\theta ,a_{1})\pi (\theta )\;d{\hat{\theta }}^{\left(2\right)}d{\hat{\theta }}^{\left(1\right)}d\theta .$$

We find the optimal combination of design parameters *a*_1_ before stage 1 and *a*_2_ before stage 2 using the backward induction principle. First we construct the Bayes optimal *a*_2_ for all possible ${\hat{\theta }}^{\left(1\right)}$ and *a*_1_. Then we construct the Bayes optimal *a*_1_ given that the optimal *a*_2_ will be used in the second stage of the trial.

##### 2.3.3.1 Optimizing the decision at the interim analysis

Denoting the marginal distribution of ${\hat{\theta }}^{\left(1\right)}$ by $f_{1}({\hat{\theta }}^{\left(1\right)},a_{1})$, we have

$$\pi (\theta )\;f_{1}({\hat{\theta }}^{\left(1\right)}|\theta ,a_{1})=f_{1}({\hat{\theta }}^{\left(1\right)},a_{1})\;\pi (\theta |{\hat{\theta }}^{\left(1\right)},a_{1}),$$

and the right‐hand side of Equation (7) can be written as

$$\int_{{\hat{\theta }}^{\left(1\right)}}f_{1}({\hat{\theta }}^{\left(1\right)},a_{1})\int_{\theta }\int_{{\hat{\theta }}^{\left(2\right)}}𝒰(\hat{\theta })f_{2}({\hat{\theta }}^{\left(2\right)}|\theta ,a_{2})\pi (\theta |{\hat{\theta }}^{\left(1\right)},a_{1})\;d{\hat{\theta }}^{\left(2\right)}\;d\theta \;d{\hat{\theta }}^{\left(1\right)}.$$

Thus, given *a*_1_ and ${\hat{\theta }}^{\left(1\right)}$, the Bayes optimal decision for the second stage is the choice of *a*_2_ that maximises

$$W_{2}(a_{2},a_{1},{\hat{\theta }}^{\left(1\right)})=\int_{\theta }\int_{{\hat{\theta }}^{\left(2\right)}}𝒰(\hat{\theta })f_{2}({\hat{\theta }}^{\left(2\right)}|\theta ,a_{1})\pi (\theta |{\hat{\theta }}^{\left(1\right)},a_{1})\;d{\hat{\theta }}^{\left(2\right)}\;d\theta .$$

For known values of ${\hat{\theta }}^{\left(1\right)}$ and *a*_1_, we can find the conditional error rates *A*_1_, *A*_2_, and *A*_12_ used in hypothesis testing in stage 2, hence we may evaluate $𝒰(\hat{\theta })$ for given *a*_1_, ${\hat{\theta }}^{\left(1\right)}$, *a*_2_, and ${\hat{\theta }}^{\left(2\right)}$. Our choices for the prior distribution and utility function mean that it is quite straightforward to compute $W_{2}(a_{2},a_{1},{\hat{\theta }}^{\left(1\right)})$ for given *a*_1_, *a*_2_ and ${\hat{\theta }}^{\left(1\right)}$. Thus, we are able to perform a numerical search seeking

$$\underset{a_{2}}{\text{argmax}}\;W_{2}(a_{2},a_{1},{\hat{\theta }}^{\left(1\right)}),$$

to find the Bayes optimal *a*_2_.

##### 2.3.3.2 Overall trial optimization

Having found the Bayes optimal parameters *a*_2_ for the second stage of the trial as a function of $\left(a_{1},{\hat{\theta }}^{\left(1\right)}\right)$, we determine *a*_1_, the Bayes optimal choice for the initial parameters, as

$$\underset{a_{1}}{\text{argmax}}\int_{\theta }\int_{{\hat{\theta }}^{\left(1\right)}}W_{2}(a_{2},a_{1},{\hat{\theta }}^{\left(1\right)})f({\hat{\theta }}^{\left(1\right)}|\theta ,a_{1})\pi (\theta )\;d{\hat{\theta }}^{\left(1\right)}d\theta .$$

We conduct a search over possible values of *a*_1_ to maximize the above integral and find the optimal choice of *a*_1_. Computing the integral for a given value of *a*_1_ by numerical integration is not straightforward. Instead, we have used Monte Carlo simulation to carry out this calculation for each value of *a*_1_.

### 2.4 Bayes optimal umbrella trials

We now consider the case of umbrella trials, where it has been argued that no multiplicity adjustment is required as the hypotheses to be tested concern different experimental treatments targeted to different molecular markers or subgroups.^28^ Since each treatment is assessed separately, an umbrella trial can be viewed a set of independent trials even though they are run under a single protocol.

We consider umbrella trials with two subgroups, as in the previous sections. However, without multiplicity adjustment, the hypothesis testing procedure reduces to testing the elementary hypotheses *H*_01_ and *H*_02_ each at level α. In applying the conditional error rate approach, only the computation of conditional error rates *A*_1_ and *A*_2_ from Equation (5) is required. Then, with $Z_{1}^{\left(2\right)}$ and $Z_{2}^{\left(2\right)}$ denoting the test statistics based on second‐stage data only, *H*_01_ is rejected if $Z_{1}^{\left(2\right)}>\Phi^{-1}(1-A_{1})$ and *H*_02_ is rejected if $Z_{2}^{\left(2\right)}>\Phi^{-1}(1-A_{2})$. No test of the intersection hypothesis is performed.

Design parameters are optimized with respect to the utility function in Equation (1). To frame the optimization problem in the same way as in the previous sections, the interim decision in a two‐stage umbrella trial will optimize only the second‐stage subgroup trial prevalences, so $a_{2}=(r_{1}^{\left(2\right)})$, while in the first stage we optimize the subgroup trial prevalences and the timing of the interim analysis, so $a_{1}=(s^{\left(1\right)},r_{1}^{\left(1\right)})$. In the case of a single‐stage umbrella trial, only the subgroup prevalences are optimized, so *a* = (*r*_1_). We have used a normal prior distribution, as defined in Equation (2), in optimizing the design parameters of single‐stage and two‐stage trials. In the case of two‐stage designs, the interim analysis uses the test statistics from the first stage and the prior distribution to perform adaptations and the final tests are performed using the conditional error rate approach.

## 3 NUMERICAL EXAMPLES AND COMPARISONS

In this section, we give numerical examples of optimized single‐stage and two‐stage designs in a range of scenarios. We show results for cases with and without multiplicity correction, referring to these as enrichment and umbrella trials, respectively. Additionally, we illustrate the optimization of the decision rule at the interim analysis. In Table 1, we provide an overview of the scenarios considered and the parameters that are optimized.

**TABLE 1:** The scenarios considered in the numerical examples. The term “opt” indicates that parameters were optimized, while “N/A” means the parameters are not applicable. The parameters $\theta_{1}$ and $\theta_{2}$ are either specified by a prior distribution in which $\psi_{1}=\psi_{2}=\psi$ or specific values of $\theta_{1}$ and $\theta_{2}$ are given

		λ	$\mu_{1}$	$\mu_{2}$	ψ	ρ	*s*^(1)^	$r_{1}^{\left(1\right)}$	$\omega_{1}^{\left(1\right)}$	$\theta_{1}$	$\theta_{2}$
Figure 2	Single‐stage	0.3	0 to 0.3	0, 0.2	0.02 to 0.44	0.5	N/A	opt	opt	prior	
Figure S2	Single‐stage	0.3	0 to 0.3	0, 0.2	0.2	−1 to 1	N/A	opt	opt	prior	
Figure 3	Interim decision	0.3	0.1	0	0.2	−0.8, 0.5	0.25, 0.5	0.3	0.3	prior	
Figure 4	Two‐stage	0.3	0, 0.3	0, 0.2	0.2	0.5	0.1 to0.9	opt	opt	prior	
Figure 5	Two‐stage	0.3	0 to 0.3	0, 0.2	0.02 to 0.4	0.5	opt	opt	opt	prior	
Figure S10	Two‐stage	0.3	0 to 0.3	0, 0.2	0.2	−0.8 to 0.8	opt	opt	opt	prior	
Figures 6 and S11	Power	0.3	0.1, 0.2	0	0.2	0.5	opt	opt	opt	0 to 0.3	0, 0.2

### 3.1 Optimal single‐stage designs

In studying the impact of the prior distribution on optimized trial design parameters $a=(r_{1},\omega_{1})$ for single‐stage designs, we consider studies where the response variance is $\sigma^{2}=1$ and the total sample size is fixed at *n* = 700. We assume a multivariate normal prior distribution for θ as defined in Equation (2) with parameters $\mu_{1}$, $\mu_{2}$, $\psi_{1}=\psi_{2}=\psi$ and ρ, and we compute optimal designs for a variety of such priors. The FWER in enrichment designs and the per‐comparison error rate in umbrella designs is fixed at α=0.05.

**FIGURE 2:**
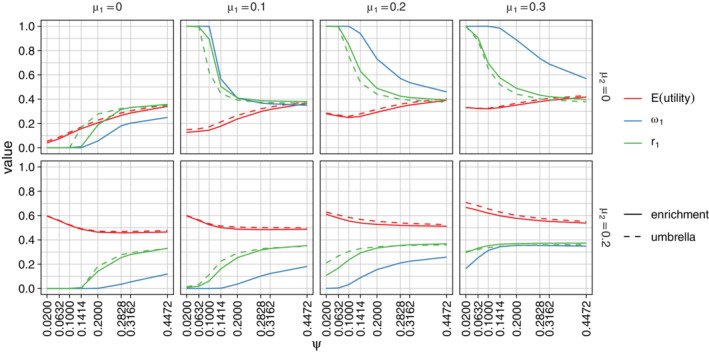
Optimized design parameters for single‐stage designs and the expected utility, averaged over the prior. Parameters are $a=(r_{1},\omega_{1})$ for enrichment trials and *a* = (*r*_1_) for umbrella trials. Results are classified by $\mu_{1}$ and $\mu_{2}$, the prior means of $\theta_{1}$ and $\theta_{2}$, and the prior SD $\psi =\psi_{1}=\psi_{2}$. The prior correlation between $\theta_{1}$ and $\theta_{2}$ is fixed at ρ=0.5 and the population prevalence of subgroup 1 is assumed to be λ=0.3 [Colour figure can be viewed at wileyonlinelibrary.com]

**FIGURE 3:**
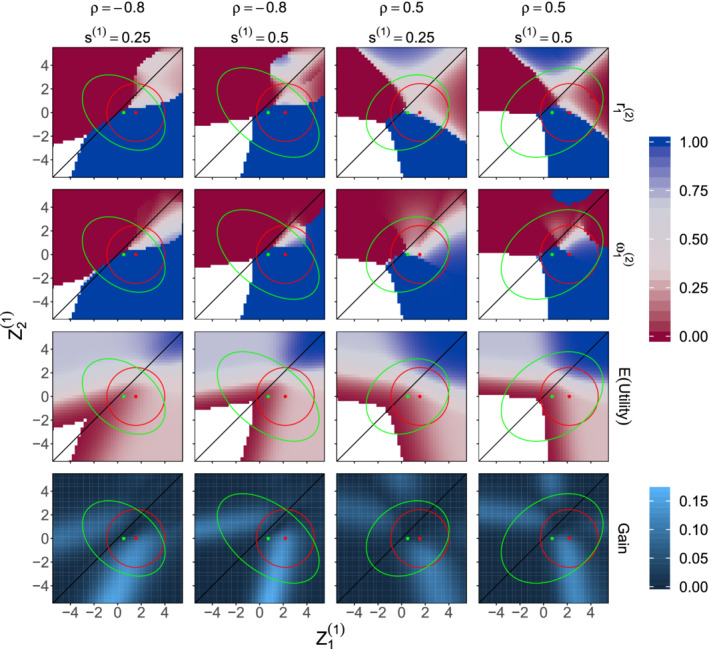
Examples of optimal adaptation rules when λ=0.3, the prior distribution for θ has parameters $\mu_{1}=0.1$, $\mu_{2}=0$, $\psi_{1}=\psi_{2}=0.2$ and ρ=0.5 or −0.8, and first stage design parameters are set as $r_{1}^{\left(1\right)}=\omega_{1}^{\left(1\right)}=0.3$ and *s*^(1)^ = 0.25 or 0.5. Optimized values of $r_{1}^{\left(2\right)}$ and $\omega_{1}^{\left(2\right)}$ are shown for each combination of first stage *Z*‐values $Z_{1}^{\left(1\right)}$ and $Z_{2}^{\left(1\right)}$. Also shown are the conditional expected utility when the trial proceeds using the optimized values of $r_{1}^{\left(2\right)}$ and $\omega_{1}^{\left(2\right)}$ and the increase in conditional expected utility compared to continuing with no adaptation. In each plot, the red circle indicates the 95% highest density region for the distribution of $\left(Z_{1}^{\left(1\right)},Z_{2}^{\left(1\right)}\right)$ when the true treatment effects are $\theta_{1}=0.3$ and $\theta_{2}=0$ and the green ellipse indicates the 95% highest density region for the prior predictive distribution of $\left(Z_{1}^{\left(1\right)},Z_{2}^{\left(1\right)}\right)$. The white regions contain values of $\left(Z_{1}^{\left(1\right)},Z_{2}^{\left(1\right)}\right)$ for which the maximum conditional expected utility is below 0.01. In these cases the numerical optimization becomes unstable and optimal values for $r_{1}^{\left(2\right)}$ and $\omega_{1}^{\left(2\right)}$ are not displayed [Colour figure can be viewed at wileyonlinelibrary.com]

**FIGURE 4:**
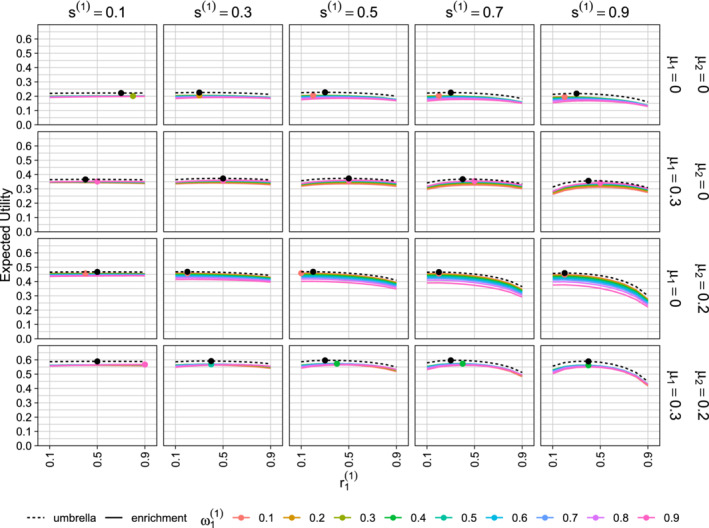
Optimization of first‐stage design parameters. The population prevalence of subgroup 1 is λ=0.3 and the prior distribution for θ has parameters $\mu_{1}=0$ or 0.3, $\mu_{2}=0$ or 0.2, $\psi_{1}=\psi_{2}=0.2$ and ρ=0.5. Each column shows results for a different value of $s_{1}^{\left(1\right)}$. The plots show the expected utility as a function of $r_{1}^{\left(1\right)}$, with coloured solid lines for different values of $\omega_{1}^{\left(1\right)}$ in an enrichment trial and black dashed lines for an umbrella trial with no multiplicity adjustment. In each panel, the colored dot indicates the combination of $r_{1}^{\left(1\right)}$ and $\omega_{1}^{\left(1\right)}$ that yields the maximum expected utility for an enrichment design and the black dot shows the optimum value of $r_{1}^{\left(1\right)}$ for an umbrella design [Colour figure can be viewed at wileyonlinelibrary.com]

**FIGURE 5:**
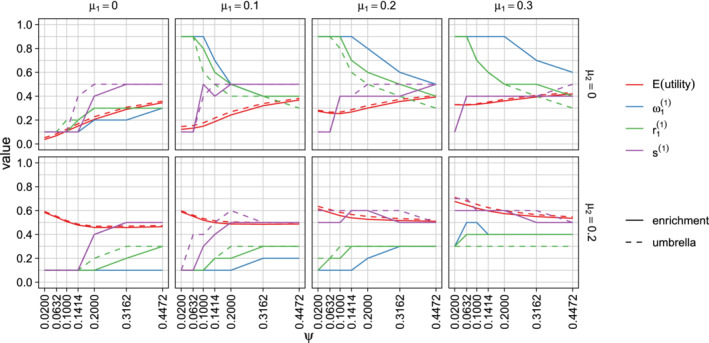
Optimized design parameters for two‐stage designs and the expected utility, averaged over the prior. Parameters are $a=(s^{\left(1\right)},r_{1}^{\left(1\right)},\omega_{1}^{\left(1\right)})$ for enrichment trials and $a=(s^{\left(1\right)},r_{1}^{\left(1\right)})$ for umbrella trials. Results are classified by $\mu_{1}$ and $\mu_{2}$, the prior means for $\theta_{1}$ and $\theta_{2}$, and by the prior SD $\psi =\psi_{1}=\psi_{2}$. The prior correlation between $\theta_{1}$ and $\theta_{2}$ is fixed at ρ=0.5 and the population prevalence of subgroup 1 is assumed to be λ=0.3 [Colour figure can be viewed at wileyonlinelibrary.com]

**FIGURE 6:**
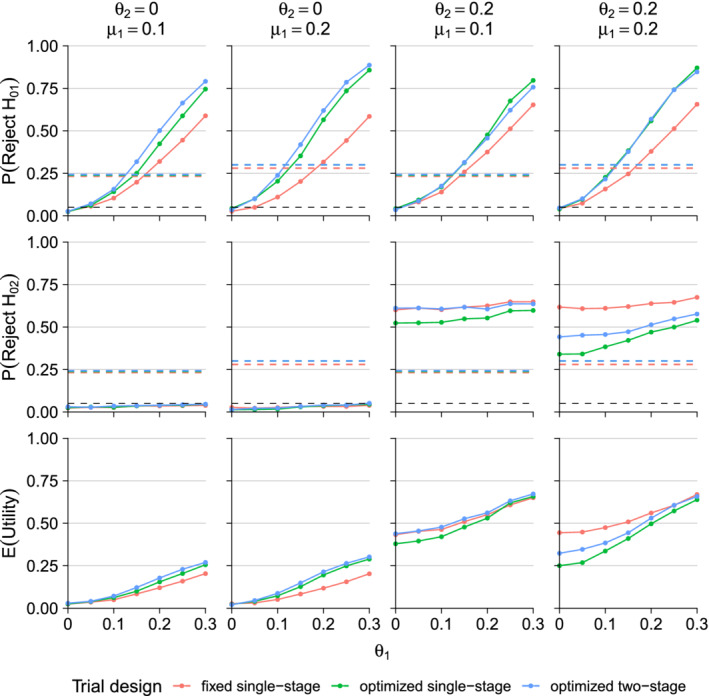
Operating characteristics of enrichment trials. The prior distribution for subgroup treatment effects $\left(\theta_{1},\theta_{2}\right)$ is normal with means $\mu_{1}=0.1$ or 0.2 and $\mu_{2}=0$, SDs $\psi_{1}=\psi_{2}=0.2$ and correlation ρ=0.5. The total sample size is 700 and the population prevalence of subgroup 1 is λ=0.3. Results are given for $\theta_{1}$ ranging from 0 to 0.3 and $\theta_{2}=0$ or 0.2. The black dashed lines in the two top rows are placed at 0.05 as reference to the significance level, while the dashed lines in the third row indicates the expected utility of the trial given the initial design parameters [Colour figure can be viewed at wileyonlinelibrary.com]

In Figure 2 we display the effect of the prior SD on the optimal design parameters when the population prevalence of subgroup 1 is λ=0.3. We considered prior SDs ψ of 0.02, 0.0632, 0.1, 0.1414, 0.2, 0.3162, and 0.44, corresponding to information from studies with 10 000, 1000, 400, 200, 100, 40 and 20 subjects in each subgroup.

The mean and variance of the prior distribution have a large impact on the optimal design parameters *r*_1_ and $\omega_{1}$. The optimal values of *r*_1_ and $\omega_{1}$ and the expected utility of the resulting designs are very similar for enrichment and umbrella designs. If $\mu_{1}>0$ and $\mu_{2}=0$, optimal values of *r*_1_ and $\omega_{1}$ are larger than 0.3, the population prevalence of subgroup 1, so the design over‐samples this subgroup. If $\mu_{1}=0$ and $\mu_{2}>0$, the optimal design under‐samples subgroup 1. When both $\mu_{1}$ and $\mu_{2}$ are greater than zero, the optimal design has *r*_1_ < 0.5 and $\omega_{1}<0.5$, reflecting the fact that it is advantageous to sample more subjects from subgroup 2 and allocate more type 1 error probability to the test of *H*_02_ since λ=0.3 implies that *P*(Reject *H*_02_) has a greater weight than *P*(Reject *H*_01_) in the utility function.

In extreme cases where $\mu_{1}=0$, $\mu_{2}\geq 0$ and the prior variance is small, the optimal design has *r*_1_ = 0, so only subgroup 2 is sampled. When $\mu_{1}>0$, $\mu_{2}=0$ and the prior variance is small, the optimal design has *r*_1_ = 1 and only subgroup 1 is sampled.

In Figure S2, we show the effect of the prior correlation ρ on the design parameters when the prior SD is ψ=0.2. We observe that the correlation has an impact on the optimal weight $\omega_{1}$ for testing the intersection hypothesis, in particular, when the treatment effects $\theta_{1}$ and $\theta_{2}$ have a high positive correlation, it is better to place most weight on one hypothesis rather than split the weight between the two hypotheses.

In Figures S3 and S4 we present further results for different values of λ, varying ρ in Figure S3 and ψ in Figure S4. Since the utility to be maximized depends on the population prevalences, the optimal design parameters vary considerably with λ. We see from Figure S3 that ρ has only a small impact on the optimal value of *r*_1_ when adjusting for multiplicity and no impact at all in umbrella designs where no multiplicity adjustment is made. Figure S4 shows that the dependence of optimal design parameters on ψ is similar to that seen in Figure 2: when the prior variance is large the optimal choices for *r*_1_ and $\omega_{1}$ are close to λ, while for smaller variances the optimal designs depend on the prior means $\mu_{1}$ and $\mu_{2}$ as well as λ.

### 3.2 Optimal two‐stage designs

Figure 3 illustrates optimal adaptation rules for two‐stage designs. In these examples *n* = 700, $\sigma^{2}=1$, the population prevalence of subgroup 1 is λ=0.3, and the prior distribution for θ has parameters $\mu_{1}=0.1$, $\mu_{2}=0$, $\psi_{1}=\psi_{2}=0.2$ and ρ=0.5 or −0.8. The first‐stage design parameters have not been optimized and are set as $r_{1}^{\left(1\right)}=\omega_{1}^{\left(1\right)}=0.3$ with *s*^(1)^ equal to 0.25 or 0.5. The FWER in enrichment designs and the per‐comparison error rate in umbrella designs is fixed at α=0.05.

The adaptation rules specify the second‐stage design parameters $a_{2}=(r_{1}^{\left(2\right)},\omega_{1}^{\left(2\right)})$ that optimize the expected utility, as defined in Equation (1), given the first stage statistics ${Z_{1}}^{\left(1\right)}$ and ${Z_{2}}^{\left(1\right)}$. The optimal $r_{1}^{\left(2\right)}$ and $\omega_{1}^{\left(2\right)}$ are calculated using the Hooke‐Jeeves derivative‐free minimization algorithm through the hjkb function in the dfoptim package^39^ in R.^40^ We also calculated the conditional expected utility if the trial continued with no adaptation, so $r_{1}^{\left(2\right)}=r_{1}^{\left(1\right)}$ and $\omega_{1}^{\left(2\right)}=\omega_{1}^{\left(1\right)}$, and the plots in the bottom row of Figure 3 show the gain in the conditional expected utility due to the optimized adaptation. In Section S3 of Appendix S1, we present optimal interim rules for further values of λ.

In Figure 4, we illustrate the procedure for optimizing first‐stage design parameters, $a=(s^{\left(1\right)},r_{1}^{\left(1\right)},\omega_{1}^{\left(1\right)})$ for an enrichment design or $a=(s^{\left(1\right)},r_{1}^{\left(1\right)})$ for an umbrella design. For each combination of prior parameters and first‐stage design parameters *a*, we generated 1000 samples of first‐stage data under treatment effects drawn from the prior distribution. For each first‐stage dataset, we found the optimal second‐stage design parameters and noted the conditional expected utility using these optimal parameters. We took the average of the 1000 values of the optimized conditional expected utility as our simulation‐based estimate of the expected utility for this choice of *a*. The optimal first‐stage design parameters for a given prior distribution are those values of *s*^(1)^, $r_{1}^{\left(1\right)}$, and in the case of an enrichment design $\omega_{1}^{\left(1\right)}$, that yield the highest expected utility.

Our results show the impact of the prior distribution on the optimized trial design parameters. The flat lines when *s*^(1)^ = 0.1 indicate that the expected utility is hardly affected by the choice of $r_{1}^{\left(1\right)}$ and $\omega_{1}^{\left(1\right)}$ when the interim analysis is performed early in the trial. When the interim analysis is performed later, the choice of first‐stage design parameters is more important. It should be noted that for each pair of prior means $\left(\mu_{1},\mu_{2}\right)$, expected utility close to the overall optimum can be achieved using a wide range of first‐stage design parameters as long as the second‐stage design is optimized, given the first‐stage data.

In Figures 5 and S10 we present optimized values of the first‐stage design parameters, *s*^(1)^, $r_{1}^{\left(1\right)}$, and $\omega_{1}^{\left(1\right)}$, given that optimal values of the second‐stage design parameters will be used following the interim analysis. The results are similar to those observed for optimal single‐stage designs. The prior variance has a large impact on the first‐stage optimal design: for smaller variances, interim analyses closer to the beginning of the trial yield a larger expected utility, while with larger variances, interim analyses after around 40% to 60% of the patients have been recruited are preferable. When the prior means are both 0 the optimal design parameters $r_{1}^{\left(1\right)}$ and $\omega_{1}^{\left(1\right)}$ are close to the subgroup 1 prevalence λ. However, if the prior suggests a benefit is more likely in subgroup 1, the optimal design over‐samples this subgroup, increasing its trial prevalence and testing weight. Figure S10 shows that, for enrichment designs, the prior correlation ρ has a large impact on the choice of $\omega_{1}^{\left(1\right)}$ but little effect on the optimal trial prevalences.

As for single‐stage designs, the optimal values of $r_{1}^{\left(1\right)}$ are similar for enrichment and umbrella designs. A notable difference is that while the prior correlation ρ has no effect at all on the optimal values of *r*_1_ in a single‐stage umbrella design, the optimal value of $r_{1}^{\left(1\right)}$ in a two‐stage umbrella design does show a small dependence on ρ. In the case of a single‐stage umbrella design, the marginal distributions of ${\hat{\theta }}_{1}$ and ${\hat{\theta }}_{2}$ do not depend on ρ and thus, with no multiplicity adjustment in testing *H*_01_ and *H*_02_, the expected value of the utility defined in Equation (1) does not depend on ρ. However, in a two‐stage umbrella trial, the optimal choice of $r_{1}^{\left(2\right)}$ and the resulting conditional expected utility depends on both ${\hat{\theta }}_{1}^{\left(1\right)}$ and ${\hat{\theta }}_{2}^{\left(1\right)}$ and it is the joint distribution of $\left({\hat{\theta }}_{1}^{\left(1\right)},{\hat{\theta }}_{1}^{\left(2\right)}\right)$, which depends on ρ, that determines the optimal value of $r_{1}^{\left(1\right)}$.

It should be noted that the procedures we have described impose a high computational burden. While it is relatively straightforward to optimize the decision at the interim analysis, the overall optimization of the trial is performed using simulations over a grid of values for the first‐stage design parameters. More rapid computation of the optimal values may be achieved by using approximations to the utility when extreme first‐stage values are observed, for example, if both $Z_{1}^{\left(1\right)}$ and $Z_{2}^{\left(1\right)}$ are large and negative, the expected utility is practically zero for all choices of $r_{1}^{\left(2\right)}$ and $\omega_{1}^{\left(2\right)}$. In practice, one may wish to add the option of stopping the trial for futility if extreme negative results are observed at the interim analysis. The methods we have presented can be extended to find efficient designs that incorporate this option by working with a utility of the form

$$\lambda \;1(\text{Reject}\;H_{01})+(1-\lambda )\;1(\text{Reject}\;H_{02})+k\;s^{\left(2\right)}\;n\;1(\text{Stop at the interim analysis}),$$

assigning a positive value *k* to each observation saved by early stopping.

### 3.3 Performance of the Bayes optimal design under specific alternative hypotheses

In this section we consider adaptive designs optimized for a particular prior distribution for $\theta =(\theta_{1},\theta_{2})$ but we evaluate their performance under specific values of θ. We consider trials with a total sample size *n* = 700, response variance $\sigma^{2}=1$, and population prevalence of subgroup 1 equal to λ=0.3. As a benchmark for comparison, we consider a nonoptimized, single‐stage design with $r_{1}=\lambda$ and $\omega_{1}=0.5$. We derive and assess the performance of single‐stage designs for which design parameters *r*_1_ and $\omega_{1}$ are optimized as described in Section 2.2, and we derive and assess two‐stage designs for which first‐stage design parameters and the adaptation rule are optimized as described in Section 2.3. In optimizing designs, we assume the normal prior distribution for θ presented in Equation (2) with $\mu_{1}=0.1$ or 0.2, $\mu_{2}=0$, $\psi_{1}=\psi_{2}=0.2$ and ρ=0.5. These priors reflects the belief that a treatment benefit is more likely in subgroup 1. The prior SD of 0.2 corresponds to information from a trial with 100 subjects in each subgroup.

We evaluate the operating characteristics of the designs for values of $\theta_{1}$ ranging from 0 to 0.3 and $\theta_{2}=0$ or 0.2. This creates scenarios with a treatment effect in only one subgroup when $\theta_{2}=0$ or with a treatment effect in both subgroups when $\theta_{2}=0.2$ and $\theta_{1}>0$. Figure 6 presents simulation results for enrichment trials and Figure S11 presents results for umbrella trials. The plots show the probabilities of rejecting *H*_01_ and *H*_02_ and the average utility at the end of the trial for a variety of combinations of $\mu_{1}$, $\mu_{2}$, $\theta_{1}$, and $\theta_{2}$. For the scenarios considered, we see that optimizing the trial for the assumed priors leads to a substantial increase in the power to reject *H*_01_ as compared to the nonoptimized, single‐stage design. However, the optimized designs have lower power to reject *H*_02_ when $\theta_{2}=0.2$. The optimized designs have a higher average utility than the nonoptimized design when $\theta_{2}=0$. If $\theta_{2}=0.2$, the two‐stage design optimized for the prior with $\mu_{1}=0.1$ has similar average utility to the the nonoptimized design but average utility of the optimized one‐stage design is a little lower; both one‐stage and two‐stage designs optimized for the prior with $\mu_{1}=0.2$ have lower average utility than the the nonoptimized design. These results are in line with previous studies^41^, ^42^ which showed adaptive enrichment designs provide the greatest advantage when a treatment effect is present in only one subgroup.

## 4 WORKED EXAMPLE: IMPLEMENTING AN OPTIMIZED ADAPTIVE ENRICHMENT TRIAL

Suppose we wish to compare an experimental treatment to a control in a phase III clinical trial. We intend to use adaptive sample allocation as there is reason to believe the new treatment may only benefit a subgroup of patients. This trial will have a normally distributed endpoint with variance $\sigma^{2}=1$ and, using information from a pilot study with 40 subjects from each subgroup, we construct a prior distribution π(θ) for the treatment effects

$$\left(\begin{array}{c} \theta_{1}\\ \theta_{2} \end{array}\right)∼N\left(\left(\begin{array}{c} 0.1\\ 0 \end{array}\right),\left(\begin{array}{cc} 0.1 & 0.05\\ 0.05 & 0.1 \end{array}\right)\right).$$

The total sample size for the trial is planned to be *n* = 700 subjects. The population prevalence of subgroup 1 is λ=0.3 and a FWER α=0.05 is to be used for the study.

Under the above assumptions, the results in Figure 5 for $\psi =\sqrt{0.1}=0.3162$ show the optimal first‐stage parameters to be *s*^(1)^ = 0.5, $r_{1}^{\left(1\right)}=0.4$ and $\omega_{1}^{\left(1\right)}=0.4$. Thus, we recruit 350 patients in the first stage of the trial with 40% of these from subgroup 1.

**FIGURE 7:**
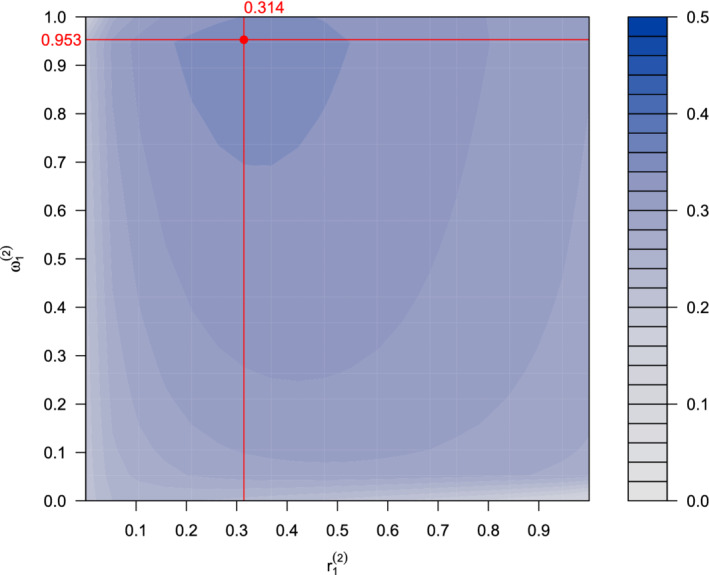
Interim optimization. The color indicates the expected utility given interim data for each combination of second‐stage prevalence $r_{1}^{\left(2\right)}$ for subgroup 1 and testing weight $\omega_{1}^{\left(2\right)}$ given the interim data [Colour figure can be viewed at wileyonlinelibrary.com]

Now suppose we observe interim estimates ${\hat{\theta }}_{1}^{\left(1\right)}=0.442$ and ${\hat{\theta }}_{2}^{\left(1\right)}=0.033$. These give *Z*‐values $Z_{1}^{\left(1\right)}=2.616$ and $Z_{2}^{\left(1\right)}=0.238$ and the conditional error rates, as defined in Equations (5) and (6), are *A*_1_ = 0.6140, *A*_2_ = 0.0184, and *A*_12_ = 0.3912. At this point, we optimize the second‐stage design parameters $r_{1}^{\left(2\right)}$ and $\omega_{1}^{\left(2\right)}$. Figure 7 plots the conditional expected utility as a function of $r_{1}^{\left(2\right)}$ and $\omega_{1}^{\left(2\right)}$ on a color‐coded scale. The maximum conditional expected utility, obtained using the Hooke‐Jeeves algorithm, is at $r_{1}^{\left(2\right)}=0.314$ and $\omega_{1}^{\left(2\right)}=0.953$. We therefore conduct the second stage of the trial using these parameter values.

Suppose, after recruiting the remaining subjects, the second‐stage estimates are ${\hat{\theta }}_{1}^{\left(2\right)}=0.272$ and ${\hat{\theta }}_{2}^{\left(2\right)}=-0.002$. The corresponding *Z*‐values are $Z_{1}^{\left(2\right)}=1.428$ and $Z_{2}^{\left(2\right)}=-0.015$, with *P*‐values $P_{1}^{\left(2\right)}=.077$ and $P_{2}^{\left(2\right)}=.506$. Since $P_{1}^{\left(2\right)}<A_{1}$ and

$$P_{1}^{\left(2\right)}<.3728=0.953\times 0.3912=\omega_{1}^{\left(2\right)}\times A_{12},$$

we can globally reject *H*_01_. However, since $P_{2}^{\left(2\right)}>A_{2}$ we cannot reject *H*_02_.

## 5 EXTENDING THE DESIGNS

The methods we have described can be extended to trial designs with more than two stages or more than two subgroups. Suppose *K* disjoint subgroups *S*_1_, … , *S*_*K*_ are specified and we wish to test the null hypotheses *H*_0*k*_: $\theta_{k}\leq 0$ against the alternatives *H*_1*k*_: $\theta_{k}>0$, where $\theta_{k}$ denotes the treatment effect in subgroup *k*. In a trial with *J* stages and a total sample size *n*, we recruit *s*^(*j*)^*n* patients in each stage, where *s*^(1)^ + ⋯ + *s*^(*J* )^ = 1, and at stage *j* we recruit $r_{k}^{\left(j\right)}s^{\left(j\right)}n$ patients from subgroups *k* = 1, … , *K*, where $r_{1}^{\left(j\right)}+\cdots +r_{K}^{\left(j\right)}=1$. The data provide estimates ${\hat{\theta }}_{1}^{\left(j\right)},\ldots ,{\hat{\theta }}_{K}^{\left(j\right)}$, at each stage *j*, from which we obtain *Z*‐values $Z_{1}^{\left(j\right)},\ldots ,Z_{K}^{\left(j\right)}$. In an enrichment design where control of the FWER is required, a suitable closed testing procedure is defined in terms of the $Z_{k}^{\left(j\right)}$. Then, *H*_0*k*_ is rejected globally at level α if all intersection hypotheses involving *H*_0*k*_ are rejected in local, level α tests.

An adaptive design can be created by repeated application of the conditional error approach. An initial reference design is stated and when adaptation occurs, the modified testing procedure is defined so as to preserve the conditional error rate of each individual and intersection hypothesis test under the updated design for the remainder of the trial. This updated design becomes the new reference design under which conditional error rates will be calculated at any subsequent adaptation point.

We can consider optimizing the choice of the design parameters *s*^(*j*)^ and $r_{k}^{\left(j\right)}$ or weights in the tests of intersection hypotheses. The generalization of our earlier approach requires a prior distribution for the treatment effects $\theta =(\theta_{1},\ldots ,\theta_{K})$ and a utility function whose expectation is to be maximised. If $\lambda_{k}$ is the population prevalence of subgroup *k*, *k* = 1, … , *K*, a natural extension of Equation (1) is

$$𝒰(\hat{\theta })=\overset{K}{\underset{k=1}{\sum }}\;\lambda_{k}\;1(\text{Reject}\;H_{0k}).$$

In Section 2.3.3 we applied backwards induction to find the optimal design for a trial with two subgroups and two stages. Since the dimension of the state space grows with the number of subgroups and stages, such a direct application of backwards induction may not be feasible more generally. Other methods of optimization can be employed to find efficient, if not globally optimal, designs. For example, in a multistage design one may construct the adaptation rule at each interim analysis assuming the trial will continue without any further adaptation. We note that the optimization process is liable to be computationally intensive and it is important to commit resources to assess trial designs in a timely manner.

## 6 DISCUSSION

We have presented a Bayesian decision theoretic framework in which a clinical trial design can be optimized when two disjoint subgroups are under investigation. Our approach has both Bayesian and frequentist elements: the rules for hypothesis testing control the type I error rate and Bayesian decision tools are used to choose the design parameters within this scheme. This allows optimization of the sampling prevalence of each subgroup and weights in a weighted Bonferroni test of the intersection hypothesis, as well as optimal adaptation of these design parameters at the interim analysis. The optimal design maximizes the expected value of the specified utility function, averaged over the prior distribution assumed for the treatment effects in the two subgroups. After focusing on two‐stage trials with two subgroups in Sections 2 and 4, we outlined how our optimization framework may be extended to allow more subgroups or stages in the trial in Section 5.

Our results provide insights into how the mean and variance of the prior distribution affects the optimal timing of the interim analysis and the trial prevalences for each subgroup of patients. In practice, it is advisable to consider the sensitivity of the design's efficiency to modeling assumptions in order to create a trial design with robust efficiency.

In contrast to adaptive enrichment designs where recruitment is either from the full patient population or restricted to a single subgroup, we propose sampling from each subgroup at a specific rate which may differ from its population prevalence. We acknowledge that achieving the optimized prevalences in a trial may be challenging: additional screening will be required and over‐sampling a particular subgroup may delay a trial compared to an all‐comers design.^43^, ^44^ If logistical considerations imply that each subgroup is either dropped or sampled according to its population prevalence, our framework can still be used to optimize the other design parameters.

In Section 3.2 we discussed designs with the option of early stopping for futility and how the utility function might be modified to facilitate optimizing such designs. A similar approach could be followed to relax the requirement of a fixed total sample size and allow re‐assessment of future sample size at an interim analysis.

We have defined methods for normally distributed observations and a normal prior for treatment effects. While this has allowed us to demonstrate how to construct such designs, it is not a necessary restriction. With normally distributed responses, one could allow a separate response variance for each patient subgroup, placing prior distributions on these variances. In trials with other types of response distribution, including survival or categorical endpoints, standardized test statistics will still be approximately normally distributed if sample sizes are large enough, although nonnormal prior distributions may be appropriate.^45^

We assumed the null hypotheses of interest are that there is no treatment effect in each subgroup. Our decision theoretic framework can accommodate other formulations, such as testing for treatment effects in the full population and in one particular subgroup,^8^, ^20^, ^22^, ^23^, ^24^, ^46^, ^47^, ^48^ in which case the stage‐wise test statistics for different subgroups are correlated. Care is required to ensure that enrichment designs control FWER when test statistics are correlated but this is not an issue in umbrella trials with separate level α tests for each null hypothesis.^31^

Although we have focused on hypothesis testing instead, estimating treatment effects after an adaptive trial is also important.^49^ Simultaneous or marginal confidence regions for parameters, with or without multiplicity adjustment, can be constructed following a two‐stage design.^50^, ^51^ Point estimates may be obtained by a weighted average of the treatment effects observed in the first and second stages^11^, ^52^ but, due to the sample size adaptations and subgroup selection these estimators may be biased with the bias depending on the specific adaptation rules and the true parameter values. A thorough investigation of estimation for adaptive enrichment designs will be a topic of future research.

Software in the form of an R package is available at https://github.com/nicoballarini/OptimalTrial.

## AUTHOR CONTRIBUTIONS

Dr Ballarini and Dr Burnett are the co‐primary authors and they contributed equally to this work.

## Supporting information

Appendix S1. Technical appendices and additional simulation results.

## Data Availability

Data sharing not applicable to this article as no datasets were generated or analyzed during the current study.
