# Supplementary figures and images for: Optimizing subgroup selection in two‐stage adaptive enrichment and umbrella designs
Source: Stat Med. 2021 Mar 29;40(12):2939–56. doi: 10.1002/sim.8949 (PMC8251960; doi:10.1002/sim.8949)

Single Stage Trial

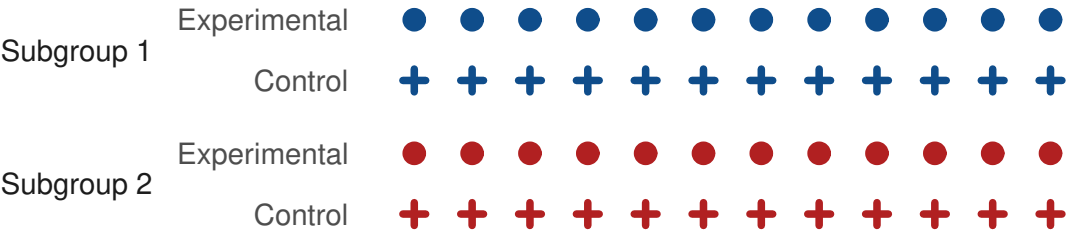

Adaptive Enrichment Trial

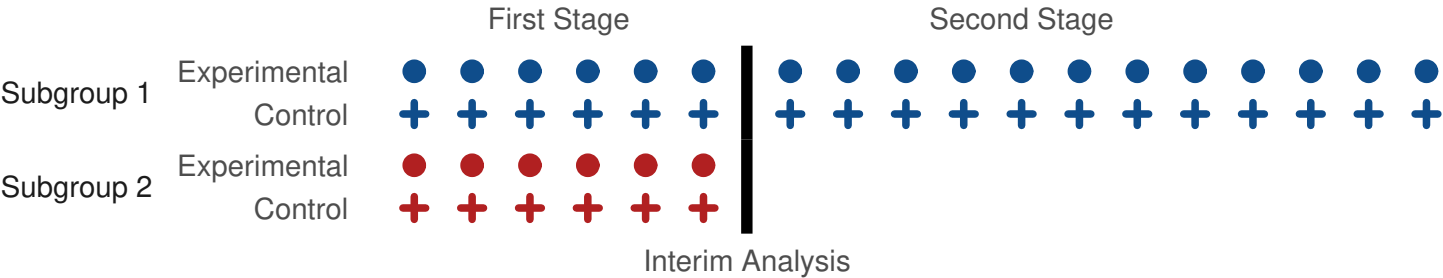

Adaptive Bayes Optimal Trial

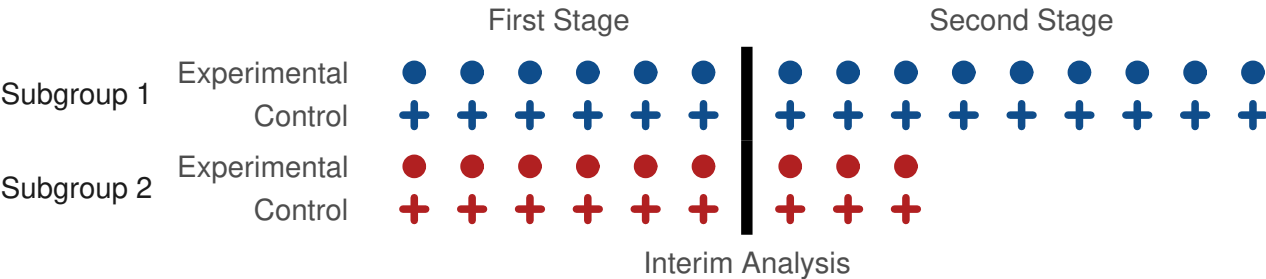

Supplement: Supplementary file 1 — Appendix S1. Technical appendices and additional simulation results. [file SIM-40-2939-s001.pdf]
